# Core Substances and Related Bio-Activities on Anti-Lung Cancer Cell A549 of *Pleione* Pseudobulb

**DOI:** 10.3390/ph19050800

**Published:** 2026-05-20

**Authors:** Chao Huang, Ge Li, Surong Li, Ruyu Yao, Angkhana Inta, Lu Gao, Lixin Yang

**Affiliations:** 1 School of Ethnic Medicine, Yunnan Minzu University, Kunming 650504, China; skt22110@outlook.com (C.H.); 17779714986@163.com (S.L.); 2 Key Laboratory of Economic Plants and Biotechnology, Kunming Institute of Botany, Chinese Academy of Sciences, Kunming 650201, China; 3 Yunnan International Joint Laboratory of Health Plant Resources Development & Yunnan Key Laboratory for Wild Plant Resources, Kunming Institute of Botany, Chinese Academy of Sciences, Kunming 650201, China; 4 Department of Biology, Faculty of Science, Chiang Mai University, 239 Huay Kaew Road, Chiang Mai 50200, Thailand; 5 Yunnan International Joint Laboratory of Southeast Asia Biodiversity Conservation, Xishuangbanna 666303, China

**Keywords:** *Pleione* Pseudobulb, Naxi traditional medicine, anti-lung cancer

## Abstract

**Background/Objectives**: The Naxi people in Northwest Yunnan of China have used alcohol-soaked *Pleione* Pseudobulbus, which is the Pseudobulbus of *Pleione bulbocodioides* Rolfe (PBR), for lung diseases and tumors for a long period of time. This study aims to explore underlying mechanisms of bioactive ingredients in PBR, as well as to underscore the synergy between traditional medicine and modern pharmacological research. **Methods**: We verified the anti-tumor effects of the PBR extract through in vitro cell experiments, and explored its underlying mechanisms by combining untargeted metabolomics with network pharmacology to predict the related targets. **Results**: The anti-tumor potential of PBR extracts was systematically evaluated by integrating chemical profiling with in vitro cell-based assays. Untargeted metabolomics tentatively annotated metabolites spanning 12 major chemical classes, several of which have been previously reported to possess anti-tumor activity. To validate these annotations, prioritized candidates were further examined by LC-MS/MS against authentic reference standards at the nanogram scale, which confirmed the presence of sclareol—a naturally occurring diterpene with documented anti-tumor properties—as a constituent of PBR. Consistent with this chemical evidence, the PBR extract exerted multi-faceted anti-tumor effects in A549 lung cancer cells: it significantly suppressed proliferation, migration, and invasion; induced G0/G1-phase cell-cycle arrest; disrupted mitochondrial membrane potential; and modulated the expression of apoptosis-related proteins. **Conclusions**: By highlighting the pharmacological properties of cultivated PBR, we identified 118 overlapping targets between PBR compounds and lung disease-related targets, and we further selected 25 core lung cancer targets with high topological importance. This study suggests that the primary active compounds of *Pleione bulbocodioides* (Franch.) Rolfe may exert anti-lung cancer activity, potentially through targeting the EGFR tyrosine kinase inhibitor resistance pathway and the PI3K-Akt signaling pathway. Furthermore, in silico molecular docking suggested that the two major active compounds exhibited favorable predicted binding affinities with four core targets, particularly EGFR and AKT1, providing a basis for further experimental validation. These results support the potential value of Naxi traditional medicine and the need to further research onthese medicinal plants, thereby promoting Chinese herb medicine conservation efforts in the Naxi region.

## 1. Introduction

The genus *Pleione*, as an important source of the traditional Chinese medicine “Shancigu,” has yielded 183 identified chemical constituents, including phenanthrenes, bibenzyls, glucosyl-benzyl succinate derivatives, flavonoids, lignans, and terpenoids. These compounds play important roles in anti-tumor, anti-neurodegenerative, and anti-inflammatory activities [1]. *Pleione bulbocodioides* (Franch.) Rolfe is one of the cultivated medicinal plants in Naxi communities of NW Yunnan. It belongs to the genus Pleione in the family of Orchidaceae. PBR is widely used in these communities to relieve cough and phlegm and to treat respiratory ailments such as cough, asthma, cold, lung fever, and other lung diseases, mainly in children and the elderly. According to the Chinese Pharmacopoeia (2020 version), PBR is associated with the liver and spleen meridians and is traditionally used for dispersing phlegm, clearing heat, and detoxification [2]. Previous phytochemical studies have reported 85 components from PBR, covering 10 chemical types, including phenanthrene, benzyl compounds, glycosides, lignans, anthraquinone, flavonoids, and steroids. Recent pharmacological experiments have shown that polysaccharides derived from shiitake mushrooms, which are present in PBR, inhibit H22 solid tumors and liver cancer in a mouse model. Shiitake mushroom polysaccharides can inhibit the proliferation of tumor cells. The inhibitory activity of compounds isolated from the autumn flower garlic orchid was tested on various tumor cell lines. One of these compounds, 10,10′-tetrahydro-2, 2′-biphenanthrene, demonstrated strong inhibitory activity against the lung cancer cell line A549 [3]. *Myrciaria dubia* exhibits anti-oxidant, anti-inflammatory, and A549 lung cancer cell proliferation-inhibiting effects [4]. The lutein and carotenoids in lettuce possess a certain degree of anti-tumor activity [5], and *Citrus bergamia* can influence lipid metabolism [6]. However, only a few studies have evaluated PBR as a promising candidate source material for anti-lung cancer activities.

Lung cancer remains a major global health burden, accounting for 11.4% of newly diagnosed cancers in 2020 and approximately 1.6 million deaths annually [7,8,9,10]. Clinically, lung cancer is classified into small cell lung cancer (SCLC) and non-small cell lung cancer (NSCLC) based on histological characteristics, with NSCLC representing approximately 85% of cases [11,12,13]. Despite advances in conventional therapies—including chemotherapy, surgery, and radiation—these approaches are often limited by severe side effects and modest efficacy, particularly in advanced NSCLC where 5-year survival rates remain critically low [14].

Growing evidence supports the therapeutic potential of natural products and traditional Chinese medicine (TCM) in cancer treatment [15,16]. Notable examples include ardisiacrispin A from *Labisia pumila*, which inhibits lung cancer development [10]; *Mitragyna speciosa* extracts, which suppress A549 cell proliferation [17]; and arenobufagin from *Bufo viridis*, which induces apoptosis and G2/M arrest in A549 cells [18]. Non-targeted metabolomics of PBR extracts have revealed multiple bioactive compounds with anti-tumor properties, including dimethylcurcumin, nitromifene, protobassic acid, flavones, cycloartocarpin, lovastatin, rosavin, annonisin, and bromelain [19,20,21,22,23,24,25,26,27]. Similarly, extracts from pomegranate peel, asparagus, carrot, and Antarctic lichen Gondwania regalis have demonstrated promising anticancer activities across various malignancies [28,29,30,31,32,33].

Network pharmacology has emerged as a powerful approach to elucidate the multi-target, multi-signaling pathway mechanisms underlying natural product efficacy. By transcending the limitations of traditional “single-target, single-drug” paradigms, this systems-level methodology aligns with TCM’s holistic principles of syndrome differentiation and treatment [34,35,36]. Integration with artificial intelligence and high-throughput technologies further enables efficient target prediction, rational drug combination design, and cost-effective drug development, facilitating TCM modernization and discovery of novel anticancer therapeutics.

PBR, widely used in Naxi medicine in NW Yunnan, has a long history of medicinal use and is currently applied to lung-related diseases in terms of our ethnobotanical research before. Building upon the traditional knowledge of the Naxi people concerning PBR processing techniques, the present study aimed to characterize the primary metabolites of PBR and to evaluate its anti-lung cancer activity. To this end, untargeted metabolomic profiling was performed to elucidate the chemical constitution of PBR, and network pharmacology analysis was subsequently integrated to delineate the putative molecular mechanisms underlying its inhibitory effects on lung cancer progression. In vitro assays employing the A549 cell line were further conducted to validate the antiproliferative activity of PBR, thereby providing experimental corroboration of its ethnopharmacological application in the management of pulmonary malignancies.

## 2. Results

### 2.1. PBR Related Traditional Knowledge

It is generally believed that whole plants and their constituent ingredients are more effective than processed plant tissues. Among the Naxi herb doctors interviewed in 12 townships of Yulong County of Lijiang City for this study, some of them believed that fresh PBR had better effects, whereas others believed that dried PBR is as effective as fresh PBR. However, due to the seasonality and difficulty of preserving plant tissues, different methods are applied for storage by Naxi herb doctors. The commonly used four processing methods are fresh, dried in the sun or shade, soaked with honey and alcohol, steamed, and then dried. Among the processing methods, all Naxi herb doctors used fresh plants, with most also using PBR processed by soaking in honey and alcohol, which is a traditional processing craft that can be stored.

Among 43 Naxi herbal doctors interviewed for this study, most of them used PBR in their formulas. According to the indication of diseases and disease classification, traditional treatment with a PBR-based formula can be divided into cancer, anti-inflammatory, respiratory system diseases, and skin and wound diseases. Thirty-three Naxi herb doctors used PBR to treat lung cancer, with PBR having low toxicity and being well tolerated by the elderly and children. At the same time, PBR can improve immunity and help to keep the lungs moist, with thirty-eight Naxi herb doctors using PBR to treat lung-related diseases. Therefore, we hypothesis that PBR processing method may involve relative bio-activities as well anti-tumor properties, providing a knowledge “prototype” and a source of medicinal materials for this study.

### 2.2. Identification of Metabolites in PBR Extracts

**Figure 1:**
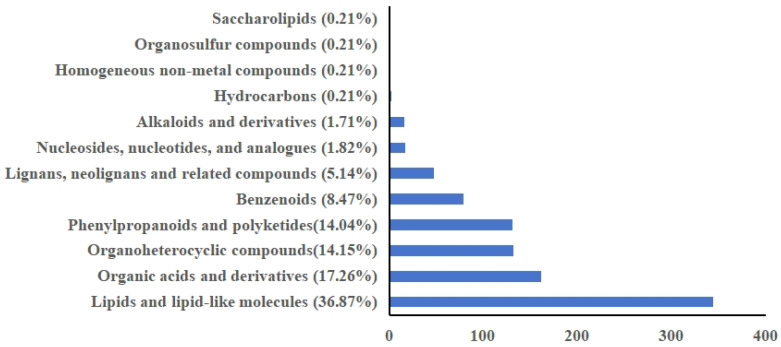
Classification of metabolites in dry PBR ethanol extracts.

**Figure 2:**
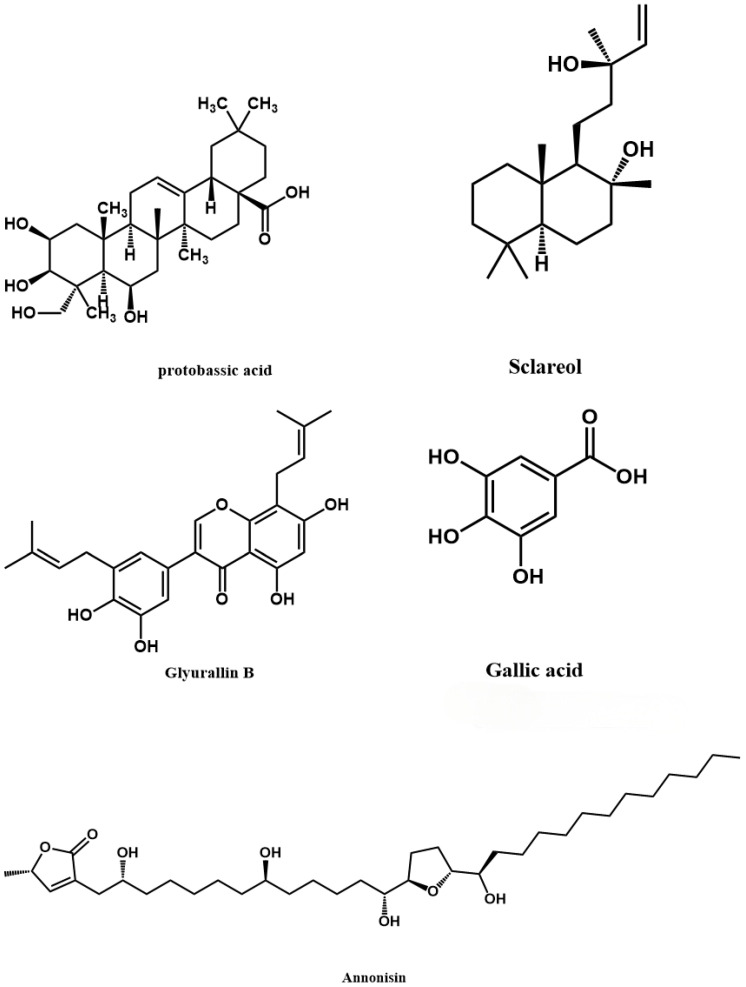
Structures of putative active compounds in PBR extracts (tentatively annotated).

We detected a total of 8510 peaks in the various ethanol extracts from PBR pseudobulbs, of which 1226 had a peak height greater than 10,000. After comparing the chemicals listed in the human metabolome database (HMDB), we obtained 938 valid peaks for classification and organization. Based on HMDB data, the potential compounds extracted from PBR pseudobulbs revealed that many of the major chemical components (with peak areas greater than 10,000) exhibit potential anti-tumor and other bioactive properties (Figure 1). The categories of compounds with the highest content in PBR pseudobulbs were lipids and lipid-like molecules, followed by organic acids and derivatives, as well as organoheterocyclic compounds, phenylpropanoids, and polyketides. Among the screened hits, annonisin is a bis-tetrahydrofuran annonaceous; as a representative of the acetogenin family, it potently inhibits mitochondrial complex I (NADH: ubiquinone oxidoreductase), depletes intracellular ATP, and elicits selective cytotoxicity against a panel of tumor cell lines including HeLa and IGROV-1 ovarian carcinoma cells [26]. Protobassic acid is a pentacyclic oleanane-type triterpene whose mono- and bidesmoside saponins (e.g., 16α-hydroxyprotobassic acid glycosides such as arganin C) exhibit potent cytotoxicity against PC-3 prostate cancer cells with IC_50_ values as low as 13.8 μM and well-defined structure–activity relationships [20]. Diosbulbin B is a furanoid norclerodane diterpene lactone isolated from *Dioscorea bulbifera* that targets the transcription factor YY1 to induce G0/G1-phase arrest and apoptosis in non-small-cell lung cancer cells (A549, PC-9, H1299) and BRAF-mutant papillary thyroid cancer cells, and suppresses xenograft tumor growth in vivo [37]. Gallic acid (3,4,5-trihydroxybenzoic acid) is a ubiquitous plant polyphenol that selectively inhibits the proliferation of hepatocellular, gastric, lung and several other carcinoma cells through ROS-dependent intrinsic apoptosis, accompanied by caspase-3/-9 activation, mitochondrial membrane potential depletion, matrix metalloproteinase inhibition and cell-cycle arrest [38]. Sclareol, a labdane-type diterpene isolated from Salvia sclarea, induces G0/G1 cell-cycle arrest, inhibits DNA synthesis, and triggers ROS-mediated apoptosis—and, more recently, ferroptosis—in leukemic, breast, and lung adenocarcinoma cells [38]. Collectively, these five chemically (tentatively annotated) diverse natural products (Figure 2)—a bis-THF polyketide acetogenin, an oleanane triterpene aglycone (with its bioactive saponins), a furanoid clerodane diterpene lactone, a low-molecular-weight trihydroxybenzoic acid, and a labdane diterpene alcohol—suppress tumor growth through complementary mechanisms encompassing mitochondrial complex I inhibition, saponin-driven membrane-active cytotoxicity, transcription factor (YY1)-mediated cell-cycle arrest and apoptosis, ROS-dependent intrinsic cell death, and DNA synthesis blockade coupled with ROS/ferroptosis induction.

### 2.3. Detection of Target Compounds in the 95% Ethanol Crude Extract of Pleione Pseudobulb

**Figure 3:**
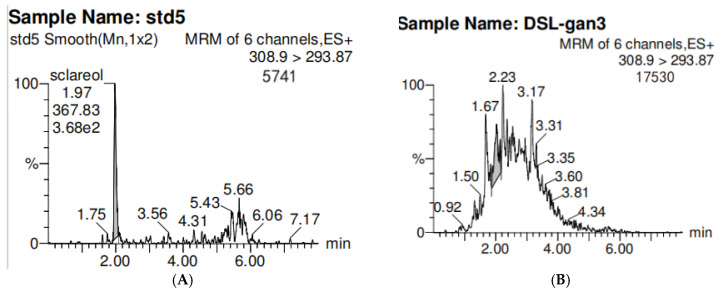
LC-MS chromatogram and mass spectrum of the target compound. (**A**) Sclareol reference standard. (**B**) Crude 95% ethanol extract of *Pleione* Pseudobulb.

LC-MS/MS analysis of the 95% ethanol crude extract of Pleione pseudobulb (PPR) was performed to experimentally validate the candidate constituents prioritized from the untargeted metabolomics dataset (Figure 3, Table 1). Among them, sclareol, a naturally occurring labdane diterpene biologically plausible in plant material, was selected for absolute quantification by LC-MS/MS against an authentic reference standard. The spectral feature attributed to sclareol displayed retention time, accurate mass, and MS/MS fragmentation in close agreement with the reference standard, thereby confirming both its identity and content in the PPR extract. Detailed chromatographic conditions, calibration data, and mass spectrometric identification parameters for sclareol are provided in the Supplementary Materials (S1).

### 2.4. Analysis of In Vitro Cell Experiments Results

#### 2.4.1. PBR Extracts Inhibit A549 Cell Growth and Migration

**Figure 4:**
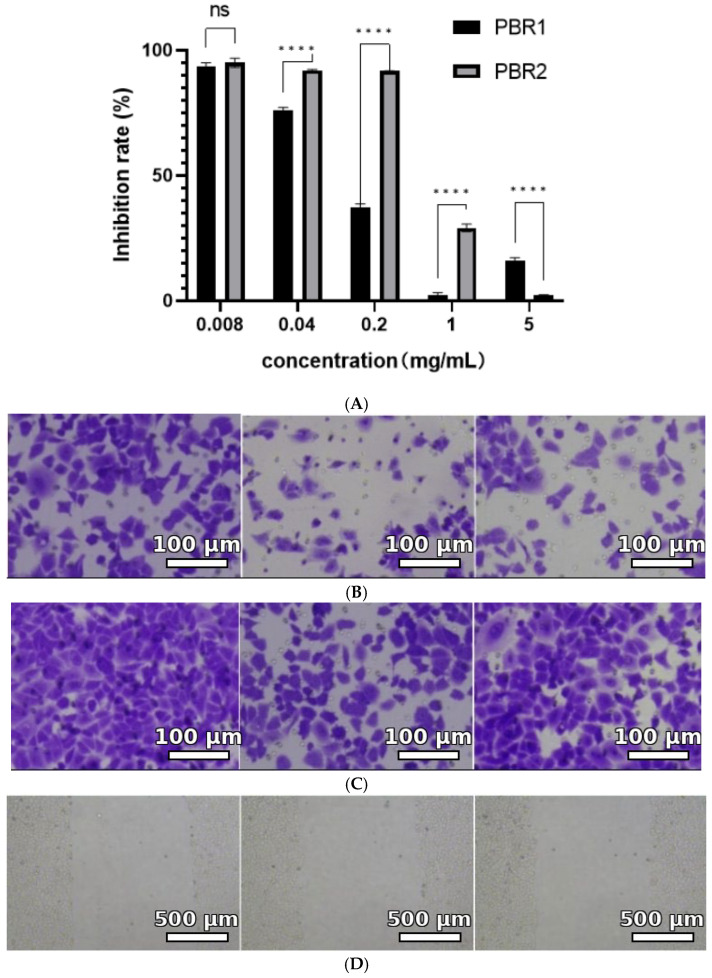

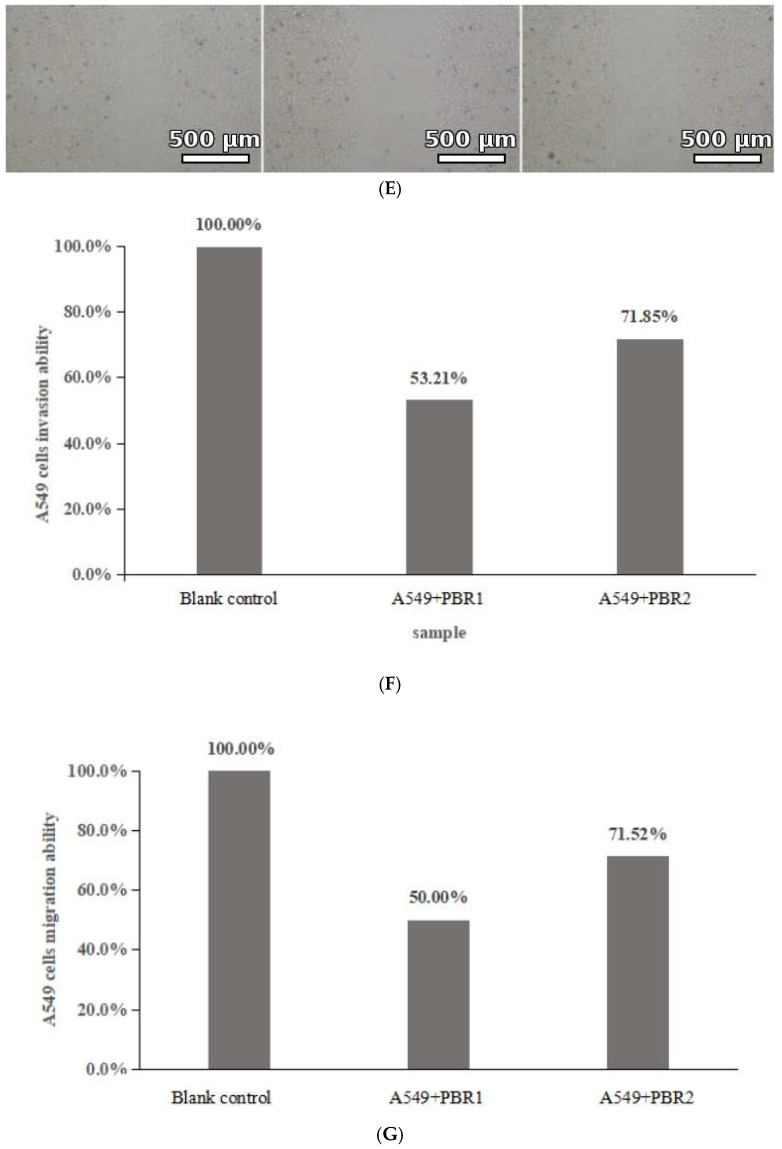
PBR extracts affect the migration and invasion of A549 cells. (**A**) Assessment of A549 cell viability as a function of PBR1 or PBR2 concentration. The MTS method was used to measure cell viability. (**B**–**E**) Visualization of cell migration and invasion ability by the Transwell crystal violet staining method. (**B**) Changes in cell invasion ability after PBR1 and PBR2 treatment. (**C**) Changes in cell migration ability after PBR1 and PBR2 treatment. (**D**,**E**) Wound-healing assay in the absence or presence of PBR extracts. The cell surface was scratched, and the healing width was measured over time by subtracting the wound width at each time point from the wound width at the 0 h time point. (**F**) PBR extracts affect cell migration outcomes. (**G**) PBR scratch test results. In the figure, “ns” indicates no significant difference between different samples at the same concentration, “****” indicates a significant difference (*p* < 0.0001).

**Figure 5:**
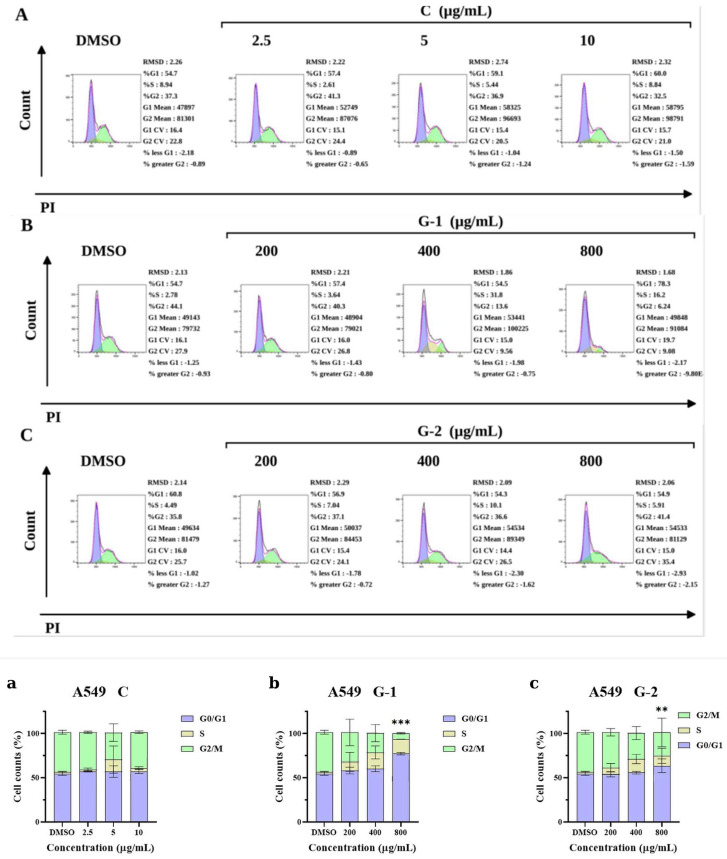
The effect of reference substance on the growth of lung cancer cells (**A**). Changes in cell-cycle progression of lung cancer A549 cells induced by PBR1 treatment (**B**). Changes in cell-cycle progression of lung cancer A549 cells induced by PBR1 treatment (**C**). Proportion of A549 lung cancer cells at different cell-cycle stages without or with PBR treatment (**a**,**b**,**c**). ** *p* < 0.01, *** *p* < 0.001.

We tested the efficacy of different ethanolic PBR extracts obtained with varying ethanol concentrations for their inhibition of cell proliferation using the human non-small cell lung cancer cell line A549. Based on the IC_50_ values of the PBR extracts, we chose the 95% ethanol extract (PBR1) and the 75% ethanol extract (PBR2) for further assays, as these two extracts showed the most significant effects among the five extract samples (Table 2). The PBR1 and PBR2 extracts both substantially inhibited the growth of A549 cells in an MTS assay (Figure 4A), with IC_50_ values of 118 μg/mL (PBR1) and 585 μg/mL (PBR2). These PBR extracts showed time- and dose-dependent inhibition of A549 cell proliferation. For PBR1, the rate of inhibition of A549 cells increased with a higher concentration, with the strongest inhibitory effect achieved at a dose of 1 mg/mL. At concentrations of 8–40 μg/mL, the PBR2 extract did not have a significant inhibitory effect on A549 proliferation. However, the inhibitory effect on A549 cells rapidly increased at concentrations of 200 μg/mL and above, demonstrating excellent inhibition of tumor cell survival. The results of the cell invasion assay are shown in Figure 5C. We established that the cell invasion ability was considerably weakened after treating lung cancer cells with each extract. After treatment with PBR1, the invasion rate decreased to 53.2% of the control (Figure 4G). Cell migration potential was similarly diminished upon treating A549 cells with either extract, with PBR1 showing a greater inhibitory effect than PBR2 (Figure 4B). Indeed, PBR1 decreased cell migration by approximately 50% following wounding, whereas PBR2 only achieved approximately 38% inhibition of cell migration (Figure 4D–F).

#### 2.4.2. PBR Extracts Induce Cell-Cycle Arrest of A549 Cells

Flow cytometry was used to analyze the cell-cycle changes of lung cancer A549 cells treated with different concentrations of PBR1 and PBR2 samples (200 μg/mL, 400 μg/mL, 800 μg/mL). The cell-cycle changes of each cycle were analyzed, and the specific experimental results are shown in the following figure (Figure 5). From the experimental results, it can be seen that with the increase in sample PBR1 and PBR2 concentrations, the proportion of G0/G1 phase cells increases, and the proportion of G2/M phase cells decreases. From different samples, at a sample concentration of 800 μ g/mL, the proportion of G0/G1 phase cells in PBR1 is greater than that in PBR2, and the number of G2/M phase cells in PBR2 is greater than that in PBR1. Based on the results, it is preliminarily speculated that the extract of *Pleione* Pseudobulb may block lung cancer A549 cells in the G0/G1 phase, thereby slowing down the occurrence of cancer. The specific anti-tumor development mechanism still needs further research.

#### 2.4.3. The Effect of PBR Extracts on Inducing Cell Apoptosis

**Figure 6:**
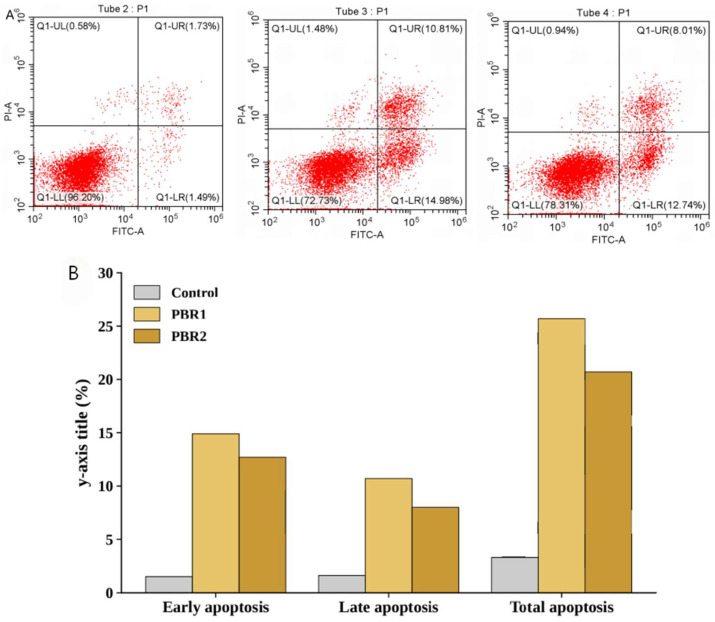
PBR extracts induce cell apoptosis. (**A**) Two-dimensional FACS profiles of A549 cells under control conditions or following PBR treatment. The proportion of cells in each quadrant is shown. (**B**) Cell apoptosis analysis. After treatment with PBR extract, the total apoptosis rate of A549 cells increased. From different periods, the extract of *Pleione* Pseudobulb increased the early apoptosis rate of cells, and PBR1 promoted A549 cell apoptosis more effectively than PBR2.

We asked whether PBR extracts might delay the development of lung cancer by promoting apoptosis. To this end, we treated A549 cells with 200 μg/mL PBR1 or 800 μg/mL PBR2 for 48 h and analyzed cells by flow cytometry. A549 cells treated with PBR1 or PBR2 showed cell apoptosis in different degrees, with PBR1-treated cells showing a substantial pro-apoptotic effect than PBR2-treated or untreated cells (Figure 6A,B). These results indicate that PBR extracts have a significant pro-apoptotic impact on A549 cells, potentially slowing down cancer progression by promoting apoptosis in cancer cells [39].

#### 2.4.4. Reactive Oxygen Species and Mitochondrial Membrane Potential

**Figure 7:**
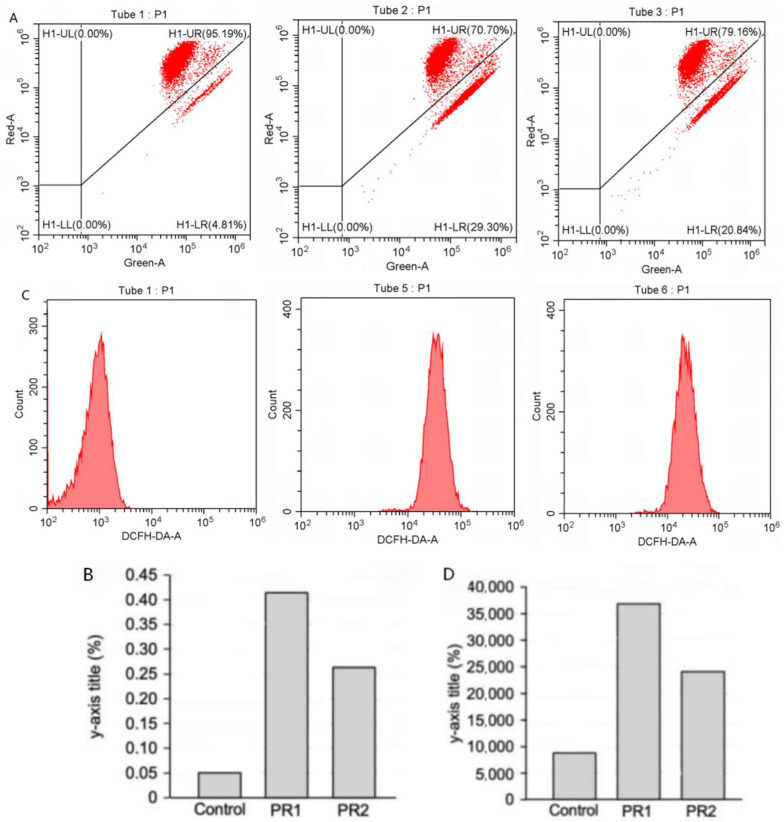
The effect of PBR extract on cellular ROS values and mitochondrial membrane potential. (**A**) FACS analysis of reactive oxygen species (ROS) accumulation in untreated A549 cells and A549 cells treated with PBR1 or PBR2. (**B**) Relative ROS levels in untreated A549 cells and A549 cells treated with PBR1 or PBR2. (**C**) FACS profiles show the mitochondrial membrane potential in untreated A549 cells and A549 cells treated with PBR1 or PBR2. (**D**) Quantification of the mitochondrial membrane potential in untreated A549 cells and A549 cells treated with PBR1 or PBR2.

To investigate the effect of PBR on reactive oxygen species (ROS) levels in lung cancer cells, we employed flow cytometry to analyze A549 cells treated with PBR1 or PBR2 for 24 h (Figure 7A,B). The mitochondria can be damaged and even dysfunctional in many human cancers [40]. The collapse of mitochondrial membrane potential is an early sign of cell apoptosis and serves as an indicator of mitochondrial function [41]. Excessive ROS levels are closely linked to mitochondrial dysfunction and apoptosis [42,43]. We observed that treatment of A549 cells with PBR1 or PBR2 extracts led to higher intracellular ROS levels, which affected mitochondrial membrane potential and altered the proliferation of A549 cells (Figure 7C,D).

#### 2.4.5. PBR Extracts Inhibit Lung Cancer Cell Development

**Figure 8:**
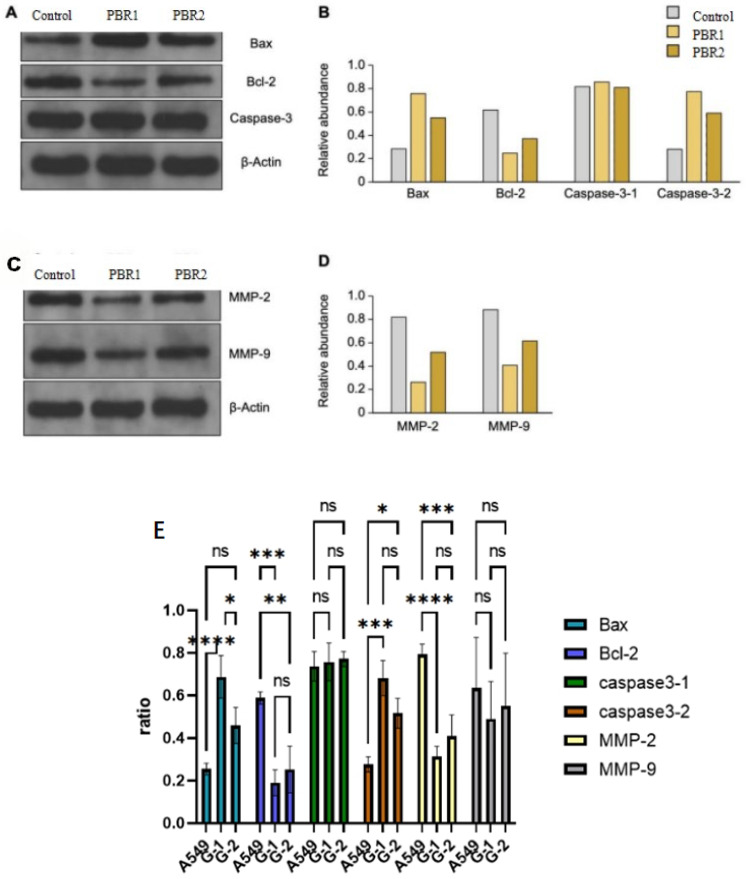
Immunoblot analysis of candidate proteins. (**A**) Immunoblot analysis of Bax, Bcl-2, and Caspase 3 abundance in untreated A549 cells and A549 cells treated with PBR1 or PBR2 for 48 h. (**B**) Quantification of protein abundance from the immunoblots is shown in (**A**). (**C**) Immunoblot analysis of MMP-2 and MMP-9 PBRotein abundance in untreated A549 cells and A549 cells treated with PBR1 or PBR2 for 48 h. (**D**) Quantification of protein abundance from the immunoblots is shown in (**C**). (**E**) ns—no statistically meaningful difference between groups; *, **, ***, ****—increasing levels of confidence that the difference is real, not due to chance; the more stars, the lower the p-value and the stronger the evidence.

Autophagy is a conserved catabolic process essential for maintaining cellular homeostasis, and dysregulated autophagy has been reported to function as an alternative cell death pathway under prolonged cellular stress or when apoptosis is impaired [44]. In the present study, network pharmacology and pathway enrichment analyses suggested that autophagy-related pathways may be among the biological processes potentially associated with the action of PBR extracts. However, as no direct autophagy markers (e.g., LC3-II, Beclin-1, or p62) were experimentally evaluated, this association remains a working hypothesis that requires further validation and is not pursued mechanistically in the present work. We focused on apoptosis, for which direct experimental evidence was obtained. TUNEL staining was used to evaluate whether PBR extracts could induce apoptosis in cancer cells. Following treatment of A549 cells with PBR1 or PBR2, the proportion of TUNEL-positive apoptotic cells increased markedly. Consistent with this, Western blot analysis revealed an increase in Bax abundance and a decrease in Bcl-2 abundance, yielding an elevated Bax/Bcl-2 ratio that favors apoptosis (Figure 8A,B). A concomitant decrease in pro-caspase-3 abundance was also observed, consistent with its proteolytic activation during apoptosis [45]. In addition, the abundances of MMP-2 and MMP-9, as well as their ratios relative to β-actin, were reduced in treated cells compared with untreated controls (Figure 8A–D), suggesting that PBR extracts may also attenuate the invasive/migratory potential of A549 cells. Taken together, these results indicate that PBR extracts exert a pro-apoptotic effect on A549 cells and may concurrently suppress matrix-degrading enzymes associated with invasion, while any contribution of autophagy remains to be experimentally verified.

### 2.5. Analysis of Network Pharmacology Results

#### 2.5.1. Intersecting Targets Between Main Bioactive Compounds and Lung Cancer

**Figure 9:**
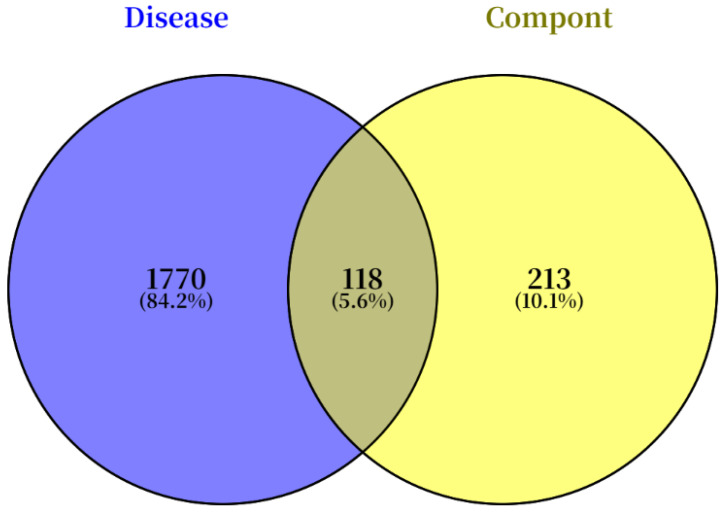
Venn diagram of compound–disease intersecting targets.

Among the metabolites of *Pleione bullbocodioides*, 16 main bioactive compounds (tentatively annotated) were screened out, primarily including terpenoids, amino acids, and other compounds. The intersecting targets were obtained by intersecting the molecular targets of these compounds with lung cancer disease targets, resulting in a total of 118 intersecting targets, as shown in Figure 9.

#### 2.5.2. Protein–Protein Interaction Network Analysis

**Figure 10:**
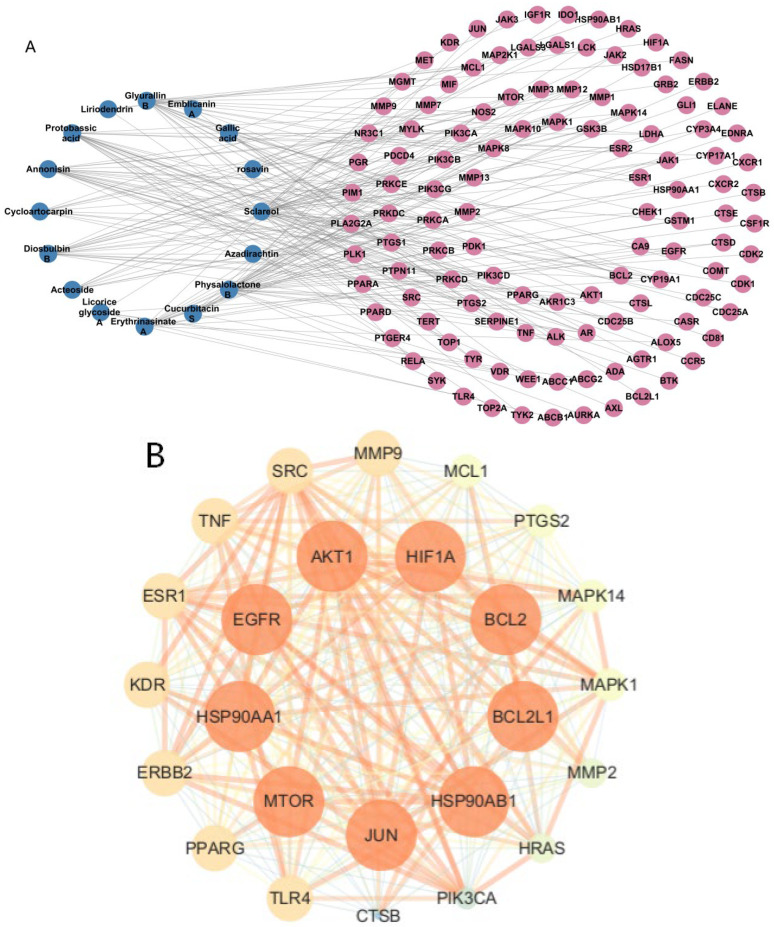
Main bioactive compounds from *Pleione bullbocodioides* and lung cancer core targets. (**A**) Compound–target network diagram: Pink represents intersecting targets between main compounds and lung cancer disease. (**B**) PPI protein–protein interaction network diagram (25 core targets).

The obtained main bioactive components and component targets were imported into Cytoscape 3.10.2 software to construct a compound–target network diagram (Figure 10A). Based on the values of degree, betweenness centrality, and closeness centrality corresponding to the targets, the main bioactive components were screened and ranked. The top 10 main bioactive compounds, including sclareol, Physalolactone B, Glyurallin B, Annonisin, protobassic acid, Diosbulbin B, Erythrinasinate A, Cycloartocarpin, Cucurbitacin S and Gallic acid, were subjected to further analysis (Table 3). Subsequently, the obtained compound–disease intersecting targets were imported into the STRING database to generate a PPI network diagram. This network diagram was imported into Cytoscape 3.10.2 software for further processing. Using Closeness > 0.002, Nearness > 187.89, and Degree > 48.45 as screening criteria, 25 lung cancer disease core targets with high degree values were screened out, and a PPI network compound diagram was constructed (Figure 10B).

#### 2.5.3. Main Functional Signaling Pathways from GO and KEGG Enrichment Analysis

**Figure 11:**
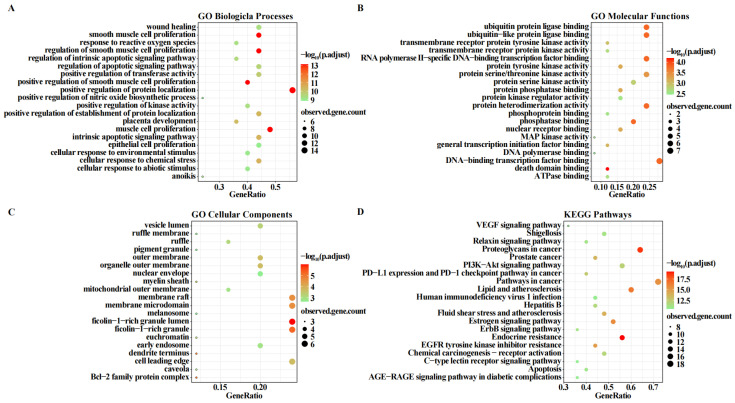
GO and KEGG enrichment analysis. (**A**) Biological process. (**B**) Molecular function. (**C**) Cellular component. (**D**) KEGG enrichment analysis.

To investigate the drug–disease mechanisms of action and signaling pathways, a total of 1495 biological processes (BPs), 52 cellular components (CCs), 79 molecular functions (MFs), and 195 KEGG signaling pathways were enriched, and enrichment analysis was performed using R software. The results showed bubble plots of the top 20 terms. Biological process (BP) analysis results showed (Figure 11A) that the synergistic effects of drugs on genes were mainly manifested in cellular sensing and response to various harmful stimuli (chemicals, radiation, oxidative stress, environmental factors) by activating protective signaling pathways to maintain cellular homeostasis. Molecular function (MF) analysis results are shown in Figure 11B, where highly enriched MFs included transcriptional regulatory functions: binding to DNA-binding transcription factors and RNA polymerase II-specific transcription factors. Protein modification and degradation functions included phosphorylation regulation, serine/threonine kinase activity and serine kinase activity, and dephosphorylation regulation: phosphatase binding and other molecular functions. Cellular component (CC) results showed (Figure 11C) that genes were mainly located in the nuclear envelope and nuclear membrane that separate the nucleus from the cytoplasm, with some participating in regulatory complexes: Bcl-2 family protein complexes to regulate apoptosis.

A total of 195 KEGG signaling pathways were enriched, with only the top 20 enrichment results displayed. The results showed (Figure 11D) that among the highly enriched KEGG signaling pathways, there were signaling pathways in cancer, EGFR tyrosine kinase inhibitor resistance pathways, PI3K-Akt signaling pathways, ErbB signaling pathways, and other apoptosis-related signaling pathways, exerting anticancer effects through multiple synergistic mechanisms of promoting apoptosis, inhibiting proliferation, and blocking carcinogenesis.

**Figure 12:**
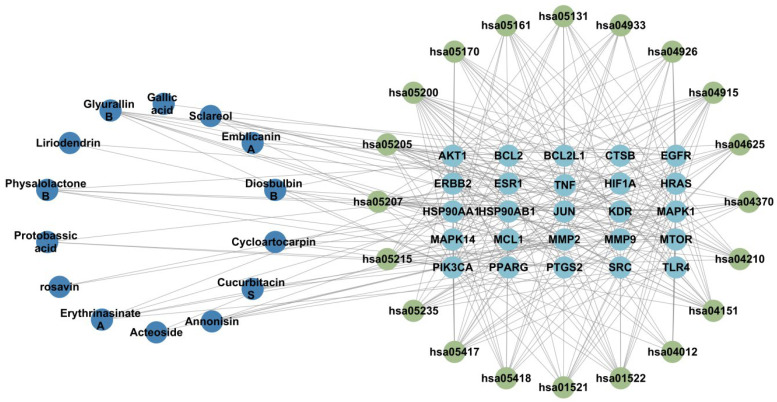
Compound–target–pathway diagram. Blue represents bioactive compounds, yellow-green represents signaling pathways, and light blue represents core targets.

The main bioactive compounds from *Pleione bullbocodioides*, 25 lung cancer disease targets, and the top 20 KEGG signaling pathways were imported into Cytoscape 3.10.2 software to further construct a compound–target–pathway diagram (Figure 12).

#### 2.5.4. Molecular Docking Results Analysis

**Figure 13:**
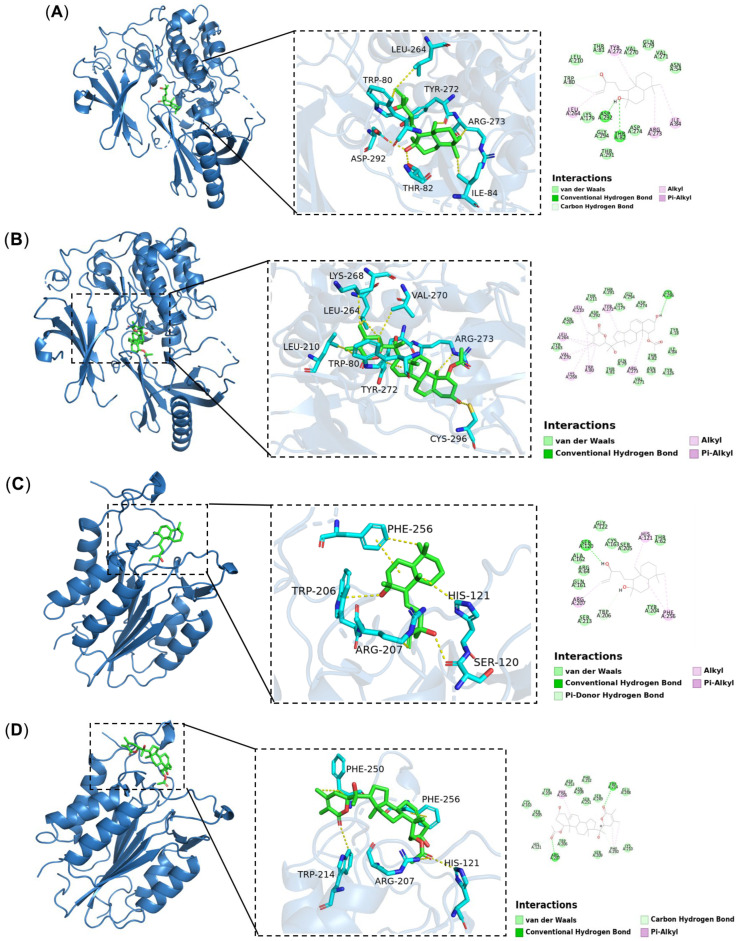

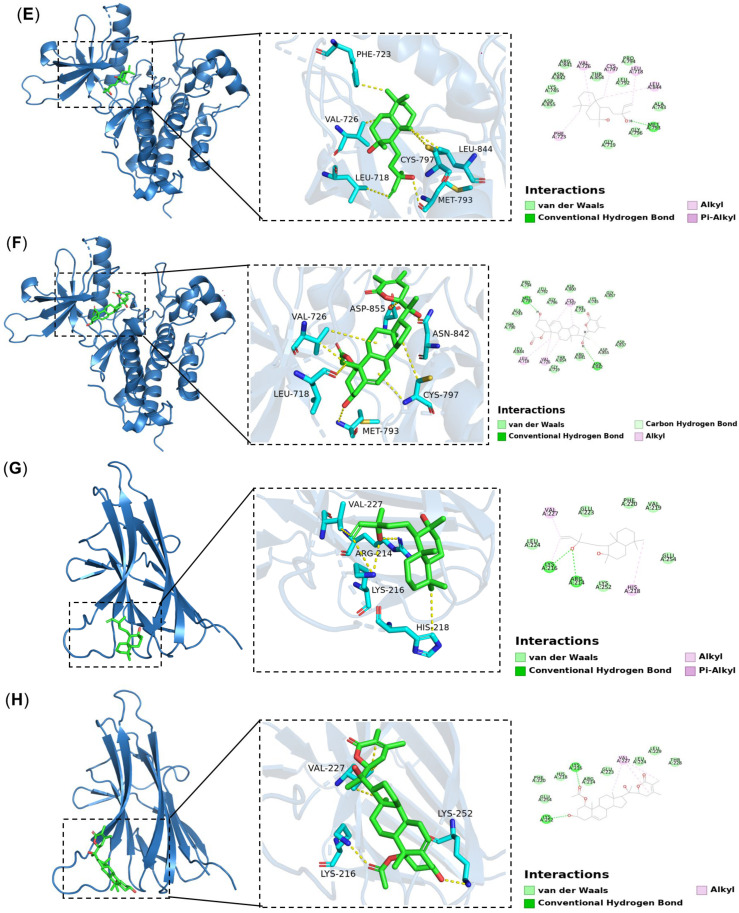
Two major active compounds (Sclareol, Physalolactone B) with four core targets (AKT1, CASP3, EGFR, TNF). (**A**) Sclareol-AKT1(PDB: 7NH5) BE: −6.9399 kcal/mol; (**B**) PhysalolactoneB-AKT1 (PDB: 7NH5) BE: −9.0437 kcal/mol; (**C**) Sclareol-CASP3 (PDB: 1CP3) BE: −6.0522 kcal/mol; (**D**) Physalolactone B-CASP3 (PDB: 1CP3) BE: −6.7965 kcal/mol; (**E**) Sclareol-EGFR(PDB: 5UGB) BE: −6.1372 kcal/mol; (**F**) Physalolactone B-EGFR (PDB5UGB) BE: 7.5049 kcal/mol; (**G**) Sclareol-TNF (PDB: 1KXG) BE: −5.3189 kcal/mol; (**H**) Physalolactone B-TNF (PDB: 1KXG) BE: −5.5547 kcal/mol.

To investigate the interactions between two major active compounds (Sclareoland Physalolactone B from *Pleione bullbocodioides* and five core proteins (AKT1, CASP3, EGFR, TNF), molecular docking was performed using MOE 2019 software. Based on the binding results, the molecular docking energies ranged from −5.3189 to −9.0437 kcal/mol. As shown in Figure 13A, on the AKT1 receptor protein, Asp292 and Thr82 formed hydrogen bonds with sclareol, while Trp80 engaged in a carbon–hydrogen bond interaction with sclareol. In addition, residues Trp80, Leu264, Arg273, Ile84, and Tyr272 established hydrophobic interactions with sclareol. In Figure 13B, Physalolactone B formed a hydrogen bond with Cys296 of AKT1 and hydrophobic interactions with residues Tyr272, Leu210, Leu264, Val270, Lys268, Trp80, and Arg273. In Figure 13C, in the CASP3 receptor, Ser120 forms a conventional hydrogen bond with sclareol, while Trp206 engages in a carbon–hydrogen bond interaction with the ligand. Additionally, residues Arg207, His121, and Phe256 participate in hydrophobic interactions with sclareol. In Figure 13D, Arg207 and Trp214 on the CASP3 protein receptor form hydrogen bonds with Physalolactone B, while His121 and Phe250 on the protein engage in carbon–hydrogen bond interactions with Physalolactone B. In addition, residues Phe256 and Phe250 form hydrophobic interactions with Physalolactone B. In Figure 13E, Met793 on the EGFR protein receptor forms a hydrogen bond with sclareol, while Val726, Cys797, Leu718, Leu844, and Phe723 on the protein engage in hydrophobic interactions with sclareol. In Figure 13F, the Met793 and Asn842 residues on the EGFR protein receptor form hydrogen bonds with Physalolactone B, while the Asp855 residue on the protein forms a carbon–hydrogen bond interaction with Physalolactone B. In Figure 13G, the Lys216 and Arg214 residues on the TNF protein receptor form hydrogen bonds with sclareol, while the Val227, Lys216, and His218 residues on the protein form hydrophobic interactions with sclareol. In Figure 13H, Lys216 and Lys252 on the TNF protein receptor form hydrogen bonds with Physalolactone B, while Val227 on the protein forms hydrophobic interactions with Physalolactone B.

## 3. Discussion

PBR (*Pleione bulbocodioides*) is an orchid species with significant medicinal value whose pseudobulbs have been widely utilized in Naxi traditional medicine as Chinese herb medicine. It demonstrates remarkable therapeutic effects, particularly in heat-clearing and detoxification, anti-inflammatory actions, and anti-tumor applications.

In this study, we demonstrated that PBR extracts can inhibit the growth and development of lung cancer cells, using the human non-small cell lung cancer cell line A549 as a model. PBR extracts affect the viability of lung cancer cells and exhibit a dose-dependent effect within a specific concentration range. Three considerations contextualize the concentrations used in our cellular assays.

First, bioactive constituents in crude botanical extracts typically represent only a small fraction of total mass; the effective concentration of active compounds is thus considerably lower than the nominal extract concentration, and the range employed here is consistent with established practice in natural product screening.

Second, direct extrapolation from in vitro concentrations to systemic exposure in vivo is constrained by pharmacokinetic factors including bioavailability, metabolism, and tissue distribution. The present concentrations are therefore most appropriately interpreted as supporting mechanistic investigation rather than predicting therapeutic dosing.

Third, crude extract assays carry inherent limitations—including compositional variability and the difficulty of attributing activity to specific constituents—that bioassay-guided fractionation and in vivo pharmacokinetic studies will need to address in future work. While direct comparison with non-malignant lung-derived cell lines was beyond the scope of the present study, several features of the dataset argue against non-specific cytotoxicity as the principal explanation for the observed effects. First, the dose–response relationship of PBR in A549 cells exhibited a well-defined sigmoidal profile with a discrete IC_50_, rather than the broadly monotonic cytotoxicity typically associated with indiscriminate cell damage. Second, PBR induced G0/G1-phase cell cycle arrest, an effect that reflects engagement of specific cell-cycle regulatory pathways rather than uniform cell death. Third, modulation of Bax, Bcl-2, and caspase-related proteins indicates activation of programmed apoptotic signaling rather than necrotic membrane disruption characteristic of non-specific cytotoxicity. Fourth, PBR concurrently inhibited migration and invasion—biological behaviors characteristic of malignant rather than non-malignant cells—indicating engagement of cancer-relevant pathways beyond simple viability reduction. Collectively, these observations are more consistent with targeted antiproliferative activity than with non-selective cytotoxicity, although formal establishment of selectivity will require direct comparison in non-malignant cell systems.

In this study, raw untargeted LC-MS data were processed through peak alignment, detection, integration, filtering, imputation, and normalization, and features were annotated against public spectral libraries (KEGG, HMDB, METLIN, LipidMaps, and MassBank). It should be noted, however, that database-only annotation in untargeted metabolomics carries an inherent risk of false-positive identification [46]. Such false positives may arise from spectral-library mismatches, since public databases co-curate compounds of human, plant, microbial, and xenobiotic origin without discriminating biological provenance; from isomer confusion, as constitutional and stereoisomers often share near-identical MS/MS spectra [47]; and from retention-time non-conformity, since library retention times transfer poorly across instruments and methods in the absence of a co-injected authentic standard [48]. To ensure the reliability of downstream analyses, all features assigned solely by spectral-library matching were uniformly designated as tentatively annotated. Among the putative constituents, Sclareol, a naturally occurring labdane diterpene, was further subjected to absolute quantification by LC-MS/MS against a commercial reference standard, with matching accurate mass, MS/MS fragmentation, and retention time, thereby providing experimental validation of its presence and content in the studied sample. Treatment with PBR extracts inhibited A549 cell proliferation by inducing G0/G1-phase [49]. Furthermore, PBR extracts modulated apoptosis-related protein expression, upregulating pro-apoptotic Bax and caspase-3 while downregulating anti-apoptotic Bcl-2. Moreover, PBR extracts elevated intracellular ROS levels, which are critically linked to mitochondrial dysfunction and apoptosis induction [50,51]. Flow cytometry and fluorescence probe analysis revealed decreased mitochondrial membrane potential following treatment, indicating that PBR extracts trigger ROS-mediated mitochondrial damage. Collectively, these findings demonstrate that PBR extracts suppress lung cancer cell growth through coordinated mechanisms: cell-cycle arrest, apoptotic signaling pathway activation, and ROS-induced mitochondrial dysfunction.

The experimental MTS assay results indicated that the samples could inhibit the progression of A549 cells. Based on the IC_50_ values PBR1 and PBR2 demonstrated strong cell growth suppression capabilities. Subsequent vitro cell experiments showed that the samples could inhibit tumor cell migration and invasion, enhance pro-apoptotic protein expression, arrest A549 cells in the G0/G1 phase, and reduce anti-apoptotic protein expression, thereby influencing cell proliferation and development.

The potential selectivity of PBR toward malignant cells also merits consideration. The IC_50_ values of PBR1 (0.118 ± 0.006 mg/mL) and PBR2 (0.585 ± 0.016 mg/mL) against A549 cells are comparable to, or lower than, those reported for the botanically related Shancigu extracts at tumor-selective concentrations [52]. The LC-MS–validated constituents sclareol has been shown to induce mitochondrial-pathway apoptosis in multiple cancer cell types with comparatively limited effects on normal cells [53,54,55]. The apoptotic signature elicited by PBR—Bax/Bcl-2 modulation, mitochondrial depolarization, ROS elevation, and G0/G1-phase arrest—closely parallels the cancer-preferential cascade previously described for these compounds, a selectivity mechanistically supported by the elevated basal ROS and narrowed anti-oxidant reserve characteristic of malignant cells. Direct validation in non-malignant lung epithelial cells (e.g., BEAS-2B) will nevertheless be pursued in future work to formally establish the therapeutic window of PBR.

Rather than representing independent phenomena, the molecular events elicited by PBR in A549 cells can be integrated into a temporally ordered, causally linked cascade centered on redox-driven intrinsic (mitochondrial) apoptosis. We propose that PBR initiates its antiproliferative action through an early elevation of intracellular ROS, which establishes a pro-oxidant intracellular milieu that exceeds the anti-oxidant buffering capacity of A549 cells. This sustained oxidative stress in turn destabilizes the outer mitochondrial membrane and promotes Bax translocation to the mitochondria while suppressing the anti-apoptotic Bcl-2, shifting the Bax/Bcl-2 balance toward a pro-apoptotic state. The resulting loss of mitochondrial membrane potential (ΔΨ_m_) is consistent with mitochondrial outer membrane permeabilization and opening of the permeability transition pore, leading to cytochrome c release into the cytosol and activation of the intrinsic apoptotic machinery, which culminates in caspase-3 cleavage—the effector step responsible for the apoptotic phenotype detected by Annexin V/PI flow cytometry.

In parallel, ROS-induced oxidative DNA damage and the bioenergetic deficit imposed by mitochondrial dysfunction compromise the energetic and biosynthetic requirements for DNA replication, accounting for the concomitant G0/G1-phase accumulation and reduction of the S-phase population. Cells that fail to complete replication under sustained oxidative stress are subsequently channeled into caspase-3-dependent apoptosis rather than re-entering the cell cycle, providing a mechanistic link between the cell-cycle and apoptotic phenotypes.

**Figure 14:**
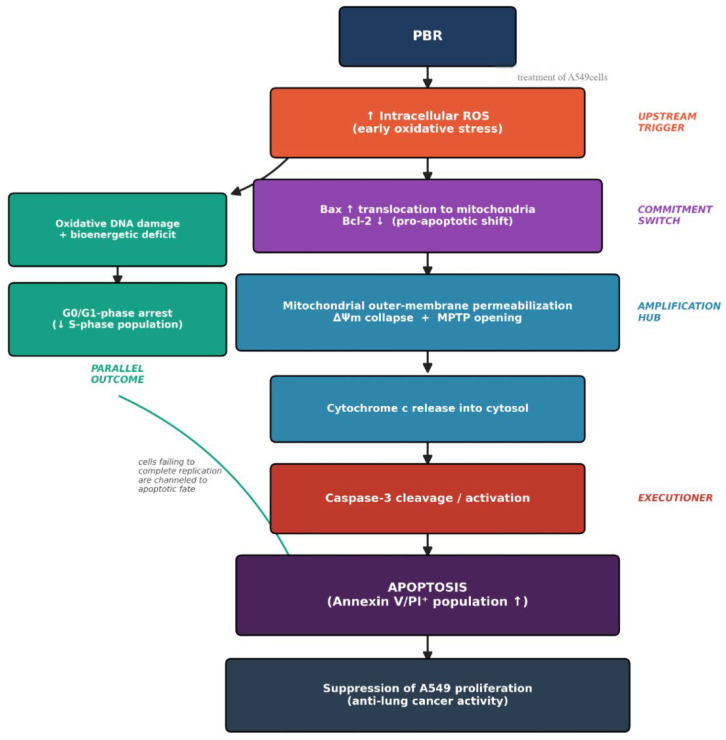
Proposed Mechanism of PBR-induced anti-lung cancer activity in A549 cells.

Within this framework, ROS elevation acts as the upstream trigger, mitochondrial membrane depolarization as the central amplification hub, Bax/Bcl-2 rebalancing as the commitment switch, caspase-3 activation as the executioner, and G0/G1 arrest as a parallel, ROS/mitochondria-dependent outcome that reinforces—rather than competes with—the apoptotic fate. This integrated ROS → mitochondrial dysfunction → Bax/Bcl-2 imbalance → cytochrome c release → caspase-3 activation → apoptosis (with parallel G0/G1 arrest) axis is fully consistent with the canonical intrinsic apoptotic pathway described for other natural product–derived anticancer agents, including sclareol and related diterpenoids [53,54,55], and provides a coherent mechanistic framework for the anti-lung cancer activity of PBR (Figure 14).

In this study, preliminary anti-tumor activity was evaluated using the MTS assay method. Furthermore, we employed its “dry–wet combined” research model through computational prediction plus experimental validation approaches to enhance reliability and translational value. We identified 118 overlapping targets between PBR compounds and lung disease-related targets through integrated network pharmacology analysis, and we further selected 25 core lung cancer targets with high topological importance using screening criteria (Closeness > 0.002, Betweenness > 187.89, Degree > 48.45). Our preliminary exploration of the mechanism underlying the anti-tumor activity of PBR extracts lays a foundation for further exploration of active anti-tumor compounds in PBR.

## 4. Materials and Methods

### 4.1. Preparation of PBR Extracts

Whole PBR plants with their root parts were collected from Ludian Village, Yulong County, Lijiang City, Yunnan Province, China, in October 2023. The species of the samples were identified by Associate Professor Lixin Yang (Key Laboratory of Economic Plants and Biotechnology, Kunming Institute of Botany, Chinese Academy of Sciences). All samples were artificially planted in Lijiang City while local staff complied with relevant national regulations. All samples were processed according to the Chinese Pharmacopoeia (2020 version) by blanching PBR pseudobulbs, using air-drying media, and physically crushing them into powder. The dried samples were extracted by incubation with different concentrations of ethanol (*v*/*v*): 95%, 75%, 55%, 35%, and 15% for 24 h at room temperature. Each extraction experiment was performed three times. The resulting solutions extracted with the same ethanol percentage were pooled, filtered, and concentrated as PBR extracts (PBR1, PBR2, PBR3, PBR4 and PBR5, respectively). Ethanol concentrations for extraction were selected based on the pharmacopeial and published literature on *Pleione pseudobulb* extraction, which predominantly employs 70–95% ethanol reflux [56,57,58].

### 4.2. Measurements and Analysis of Metabolite Contents

Metabolomics analysis of the extracts was performed using a liquid chromatography system (Waters ACQUITY UPLC I-Class) equipped with a mass spectrometry system (Xevo G2-XS QTOF) (Waters Corporation, Manchester, UK). Analysis was performed under the following conditions: flow rate, 0.4 mL/min; solvent A, 0.1% (*v*/*v*) HCOOH in water; solvent B, acetonitrile; chromatography column, Waters ACQUITY UPLCr BEH C18 1.7 μm(Waters Corporation, Milford, MA, USA); column temperature, 35 °C; analysis time, 25.00 min. Mass spectrometry conditions were as follows: when operating in the positive ion mode of the electrospray ionization (ESI) ion source, the capillary voltage was set to 2.5 kV, the desolvation temperature was maintained at 450 °C, and the flow rate of the desolvation gas (N_2_) was 900 L/h; when operating in the negative ion mode of ESI, the capillary voltage was set to 2.0 kV, the desolvation temperature was maintained at 450 °C, and the flow rate of the desolvation gas (N_2_) was 900 L/h. The mass scan range was *m*/*z* 100–1500, and the acquisition mode was MS^E^ in sensitivity mode. Data were analyzed using Masslynx 4.2 (996) software (Waters Corporation, Milford, MA, USA) [33].

Masslynx software (version 4.2, Waters Corporation, Milford, MA, USA) was used to collect raw mass spectrometry data. Progenesis QI software (version 3.0, Nonlinear Dynamics, Newcastle upon Tyne, UK; a Waters company) was used to perform peak alignment, peak recognition, normalization, and deconvolution analysis of the raw data before identifying metabolites and annotating them based on the human metabolome database (HMDB, https://hmdb.ca/) (accessed on 4 August 2025). EZinfo software (version 3.0.3, Umetrics, Umeå, Sweden) was used to perform principal component analysis (PCA) and partial least squares discriminant analysis (PLS-DA) of the above metabolome data to detect differences between experimental groups and intra-group repeatability.

### 4.3. LC-MS/MS Analysis

Chromatographic separation was performed on a Waters Acquity UPLC system equipped with a BEH C18 column (1.7 μm). The mobile phase consisted of water (A) and acetonitrile (B) with a gradient elution as follows: 0–5 min, 10% → 100% B; 5–6 min, 100% → 10% B; 6–8 min, 10% B. The flow rate was 0.3 mL/min and the injection volume was 1.0 μL.

Mass spectrometric detection was conducted on a Waters Xevo TQ-S triple quadrupole instrument operating in positive electrospray ionization (ESI+) mode. Source parameters were set as follows: capillary voltage, 3.2 kV; source temperature, 150 °C; desolvation temperature, 200 °C; cone gas flow, 150 L/h desolvation gas flow, 550 L/h. Analytes were quantified using multiple reaction monitoring (MRM).

### 4.4. Cell Cultures and Treatments

The human non-small cell lung cancer cell line A549 (RRID:CVCL0023) was purchased from the ATCC, derived from human lung tissue, was used in this study for research purposes only and cultured in RMPI1640 medium containing 10% (*v*/*v*) fetal bovine serum. Each well of a six-well culture plate was seeded with A549 cells that were in the logarithmic growth phase and showed good growth at a cell density of 2 × 10^5^ cells per well. The plates were incubated overnight at 37 °C, in 5% (*v*/*v*) CO_2_. The cells were divided into groups and subjected to the indicated treatment for 24 h [59,60,61].

### 4.5. In Vitro Cell Experiments

#### 4.5.1. Cell Viability Assay

A549 cells were inoculated with 3000 cells per well into a 96-well plate, with a volume of 100 μL per well, and the cells were cultured 12–24 h in advance, then seeded into 96-well plates and treated with different concentrations of PBR extracts for 48 h. The 3-(4,5-dimethylthiazol-2-yl)-5(3-carboxymethoxyphenyl)-2-(4-sulfopheny)-2H-tetrazolium (MTS) method was used to determine the cell inhibition rate. MTS is an analog of MTT. Absorption values at 492 nm were recorded using a multifunctional microplate reader (MULTISKAN FC). The cell viability and inhibition rate were calculated using the two-point method (Reed and Muench method: Cell viability value = (total absorbance (experimental group) − total absorbance (control group))/total absorbance (control group) × 100%).

#### 4.5.2. Wound Healing Assay

A549 cells were seeded into sterile six-well plates at a starting cell density of 1.2 × 10^6^ cells/well and cultured overnight in an incubator (37 °C and 5% CO_2_). After the PBR1 (200 μg/mL) or PBR2 (800 μg/mL) extracts were added to the wells, the plates were incubated at 37 °C and 5% (*v*/*v*) CO_2_ under saturated humidity overnight. When the cell density reached over 90%, a straight line was drawn onto the cell surface with the tip of a sterile pipette. Photographs were taken of the wound under an inverted research microscope immediately upon injury (time 0 h) and at 24 h after injury.

#### 4.5.3. Transwell Invasion Assay

After treatment with PBR1 (0 or 200 μg/mL) or PBR2 (0 or 800 μg/mL) for 24 h, the cells were transferred to Transwell plates, following the operating requirements. For the assay, 800 μL of 10% (*v*/*v*) FBS Ham’s F-12K medium (containing dual antibodies) was added into the Transwell chamber. Then, 100 μL of Matrigel was added vertically to the bottom of the upper chamber in the center of the Transwell chamber at a final concentration of 1 mg/mL and allowed to sit at 37 °C for 1 h. After Matrigel incubation, 200 μL of cell suspension from each well of the six-well plate was added to the upper chamber of the Transwell chamber for 1 h. After incubation at 37 °C in 5% (*v*/*v*) CO_2_ for 24 h, the cells on the surface of the filter screen were removed. The filter was fixed with 4% (*w*/*v*) paraformaldehyde, and the cells were stained with 0.5% (*w*/*v*) crystal violet. After staining, three fields of view were selected and observed under an optical microscope (Ta2-FL, Nikon Corporation, Tokyo, Japan).

#### 4.5.4. Cell Migration Assay

A549 cells were seeded onto a six-well plate at a starting cell density of 2 × 10^5^ cells/well and cultured overnight in an incubator at 37 °C with 5% (*v*/*v*) CO_2_. The PBR1 (200 μg/mL) or PBR2 (800 μg/mL) extracts were added, followed by cultivation under the same conditions as above for 24 h. Photographs were taken before and 24 h after treatment.

#### 4.5.5. Cell-Cycle Analysis

A549 cells were seeded onto a six-well plate at a starting cell density of 2 × 10^5^ cells/well and cultured overnight in an incubator at 37 °C with 5% (*v*/*v*) CO_2_. The PBR1 (200 μg/mL) or PBR2 (800 μg/mL) extracts were added, followed by cultivation under the same conditions as above for 48 h. Cell-cycle analysis was performed by flow cytometry (BECKMAN, CytoFLEX, Brea, CA, USA).

#### 4.5.6. Cell Apoptosis Detection

A549 cells were seeded onto a six-well plate at a starting cell density of 2 × 10^5^ cells/well and cultured overnight in an incubator at 37 °C with 5% (*v*/*v*) CO_2_. The cells were treated with PBR1 (0 or 200 μg/mL) or PBR2 (0 or 800 μg/mL) extracts and returned to the incubator for another 24 h. For detection of cell apoptosis, a 500 μL binding buffer was added to the cells according to the instructions of the kit (QS-S306, QiSai organism, MA, USA). After the cells were resuspended, 5 μL annexin V-FITC and 5 µL propidium iodide (PI) were added, followed by incubation at room temperature in the dark for 5 min. A549 cells that did not receive annexin V-FITC or PI were used as a control. Flow cytometry (CytoFLEX, BECKMAN, Brea, CA, USA) was used for the detection of cell apoptosis [59].

#### 4.5.7. Mitochondrial Membrane Potential Assay

A mitochondrial membrane potential detection kit (Qisai Biotech, Shanghai, China) was used to detect mitochondrial membrane potential according to the kit instructions. The JC-1 probe was used for staining, and the cells were incubated at 37 °C for 20 min in a cell culture incubator. After staining, the cells were washed with buffer solution (1XPBS) and analyzed by flow cytometry.

#### 4.5.8. Detection of Reactive Oxygen Species

A549 cells were seeded onto a six-well plate at a starting cell density of 2 × 10^5^ cells/well and cultured overnight in an incubator at 37 °C with 5% (*v*/*v*) CO_2_. Cells were treated with PBR1 (200 μg/mL) or PBR2 (800 μg/mL) extracts and incubated for 24 h before being stained by incubation with 10 μM DCFH-DA at 37 °C for 20 min. The cells were washed with serum-free medium three times and centrifuged at 1200 rpm for 5 min. The supernatant was discarded, and the cells were resuspended in phosphate-buffered saline (PBS) and subjected to flow cytometry.

#### 4.5.9. Western Blot Analysis

A549 cells were seeded onto six-well plates at a starting cell density of 1.2 × 10^4^ cells/well and were treated with PBR1 (0 μg/mL or 200 μg/mL) or PBR2 (0 μg/mL or 800 μg/mL) for 24 h. Total proteins were extracted from cells using RIPA buffer containing protease inhibitors. The protein concentration was assessed using a Pierce BCA kit. Subsequently, each protein sample was separated using 8–12% (*w*/*v*) SDS-PAGE, and the proteins (Table 4) were transferred to nitrocellulose membranes. After blocking with 5% (*w*/*v*) skim milk, the membranes were incubated with the following primary antibodies overnight at 4 °C. The membranes were then treated with a horseradish peroxidase conjugated secondary antibody for 1 h at room temperature. An Image Pro Plus instrument was used to capture signals [18].

### 4.6. Network Pharmacology

#### 4.6.1. Screening of Bioactive Compounds and Lung Cancer Therapeutic Targets

Based on untargeted metabolomics analysis of crude extract from *Pleione bullbocodioides*, among the metabolites detected and analyzed, 33 bioactive compounds with potential anti-lung cancer activity were screened using active as screening criteria. The molecular targets of these bioactive compounds were obtained through Swiss Target Prediction (http://www.swisstargetprediction.ch/) (accessed on 20 August 2025).

#### 4.6.2. Targets for Lung Cancer

Using the GeneCards human disease gene database (https://www.genecards.org/) (accessed on 22 August 2025), searches were conducted with “lung cancer” as the keyword, and results were sorted by score from high to low to screen the top-ranked disease targets. OMIM (https://www.omim.org/) (accessed on 23 August 2025) was further utilized to search and supplement lung cancer disease gene targets. The obtained disease targets were merged and organized, and Venn diagrams were constructed to identify the intersecting targets between bioactive compounds and lung cancer disease.

#### 4.6.3. Construction of Protein–Protein Interaction Network (PPI Network)

The intersecting targets between the main bioactive components of *Pleione bullbocodioides* and lung cancer disease were organized. Using the online STRING database (https://cn.string-db.org/) (accessed on 24 August 2025) with humans as the reference species, a protein–protein interaction (PPI) network of target genes was constructed. The network was imported into Cytoscape 10.3.2 analysis software, and using Closeness > 0.002, Betweenness > 187.89, and Degree > 48.45 as screening criteria, the main compounds of *Pleione bullbocodioides* with anti-lung cancer effects and their corresponding core targets in lung cancer disease were screened out, and a PPI network diagram was constructed.

#### 4.6.4. GO Enrichment and KEGG Analysis

The screened core disease targets were imported into the DAVID database (https://davidbioinformatics.nih.gov/) (accessed on 25 August 2025) with human species selected to obtain corresponding biological process (BP), cellular component (CC), molecular function (MF) information and KEGG signaling pathways. The top 20 GO terms and the top 20 KEGG signaling pathways were respectively selected for GO and KEGG enrichment analyses. The ggplot2, openxls, and tidyverse packages in R software (R version 4.4.0) were used for the visualization of GO and KEGG signaling pathway enrichment analysis. Finally, using the obtained top 20 KEGG signaling pathway information combined with the main bioactive components of *Pleione bullbocodioides* and lung cancer disease core targets, a compound–target–pathway diagram was constructed through Cytoscape 10.3.2.

#### 4.6.5. Molecular Docking

The 2D structures of the two main active compounds (sclareol and Physalolactone B) were obtained from the PubChem database (http://pubchem.ncbi.nlm.nih.gov/) (accessed on 26 August 2025). The 2D structures were then imported into ChemOffice 20.0 software to generate their corresponding 3D structures, which were saved as mol2 files. Subsequently, the RCSB PDB database (http://www.rcsb.org/) (accessed on 28 August 2025) was utilized to screen for protein targets with high-resolution crystal structures to serve as molecular docking receptors. PyMOL 2.6.0 software was employed to process the proteins by removing water molecules, phosphate groups, and other moieties, and the processed structures were saved as PDB files. Molecular Operating Environment 2019 software was used to perform energy minimization of the compounds, preprocess the target proteins, and identify active binding sites. Finally, MOE 2019 was executed to conduct molecular docking with calculation runs set to 50 iterations. The binding activity between the compounds and targets was evaluated based on binding energy values, and the results were visualized using PyMOL 2.6.0 and Discovery Studio 2019 software.

### 4.7. Statistical Analysis

All data are presented as means ± standard deviation (SD) from three independent experiments. Statistical comparisons between the two groups were performed using a *t*-test in Excel 2019. A *p*-value <0.05 was considered statistically significant.

## 5. Conclusions

This study offers preliminary validation of the traditional folk applications of PBR and provides evidence and theoretical support for some of its medicinal efficacy. The findings indicate that cultivated *P. bulbocodioides* has strong pharmacological values, particularly in inhibiting tumor cell growth, migration, and invasion, while also promoting apoptosis-related protein expression. Network pharmacology analysis identified several significantly enriched KEGG signaling pathways, including lung cancer-related signaling pathways, EGFR tyrosine kinase inhibitor resistance pathways, and other apoptosis-associated signaling pathways. Collectively, these signaling pathways contribute to the anticancer activity of PBR through three key approaches: (1) inducing apoptosis, (2) suppressing proliferation, and (3) inhibiting oncogenic processes. Moreover, based on both traditional use and experimental tests, PBR exhibits promising anticancer activity. Further targeted LC-MS validation confirmed the presence of sclareol corroborating the preliminary predictions and substantiating their potential anti-lung cancer activity, thus warranting further mechanistic investigation. Meriting further exploration as a potential anti-tumor agent. Future research will concentrate on identifying, quantifying, and characterizing the bioactive compounds responsible for its anti-tumor effects, as well as elucidating the underlying molecular mechanisms and signaling pathways. This foundational work will facilitate the subsequent isolation of natural anti-tumor compounds from PBR and their potential application in cancer treatment. This study offers preliminary scientific insights into the traditional uses of PBR, bridging ethnopharmacological knowledge with early-stage pharmacological exploration, and may provide a foundation for future investigations aimed at identifying candidate compounds for anticancer research.

## Figures and Tables

**Table 1:** Compound contents in the 95% ethanol crude extract of PPR.

Compound Name	Sclareol
*w*/*w*	9.38 × 10^−3^ *

Note: Values are presented as mean ± SD (*n* = 3). Statistically significant differences are indicated by * (*p* < 0.05).

**Table 2:** List of PBR extracts obtained with different ethanol concentrations.

Sample No.	Ethanol Concentration	IC_50_ ± SD (mg/mL)
PBR5	15%	2.232 ± 0.023
PBR4	35%	2.556 ± 0.058
PBR3	55%	2.099 ± 0.016
PBR2	75%	0.585 ± 0.016
PBR1	95%	0.118 ± 0.006

**Table 3:** Top 10 bioactive compounds among 16 metabolites (tentatively annotated) from *Pleione bullbocodioides*.

Compound Name	Degree	Betweenness	Closeness
Sclareol	31.0	4804.028	0.2250423
Physalolactone B	30.0	4244.423	0.22056384
Glyurallin B	23.0	3991.83	0.21626016
Annonisin	21.0	2558.0232	0.2141707
Protobassic acid	21.0	3326.7864	0.21486267
Diosbulbin B	18.0	2257.4744	0.20879121
Erythrinasinate A	15.0	2149.2559	0.20121029
Cycloartocarpin	10.0	531.11945	0.19880418
Cucurbitacin S	9.0	630.03735	0.19645494
Gallic acid	8.0	1188.5424	0.19136691

**Table 4:** Test protein-related information.

Protein Name	Manufacturer	Item Number
Bax	Wuhan Sanying Biotechnology Co., Ltd. (Wuhan, China)	D220073
Bcl-2	Wuhan Sanying Biotechnology Co., Ltd.	12789-1-AP
Caspase3	Wuhan Sanying Biotechnology Co., Ltd.	66470-2-IG
MMP-2	Wuhan Sanying Biotechnology Co., Ltd.	BS-4599R
MMP-9	Wuhan Sanying Biotechnology Co., Ltd.	10375-2-AP

## Data Availability

The original contributions presented in this study are included in the article/Supplementary Material. Further inquiries can be directed to the corresponding authors.

## Supplementary Materials

The following supporting information can be downloaded at: https://www.mdpi.com/article/10.3390/ph19050800/s1; File S1: Determination of Sclareol Content in PBR1 Samples by LC-MS.

## Abbreviations

ROS, Reactive Oxygen Species; PBR, *Pleione bulbocodioides* (Franch.) Rolfe; MTS, 3-(4,5-dimethylthiazol-2-yl)-5(3-carboxymethoxyphenyl)-2-(4-sulfopheny)-2H-tetrazolium; WB, Western Blot.
