# Supplementary material for: Core Substances and Related Bio-Activities on Anti-Lung Cancer Cell A549 of Pleione Pseudobulb
Source: Pharmaceuticals (Basel). 2026 May 20;19(5):800. doi: 10.3390/ph19050800 (PMC13210126; doi:10.3390/ph19050800)
Supplement: Supplementary file 1 [file pharmaceuticals-19-00800-s001.zip › pharmaceuticals-4319614-supplementary.pdf]

Quantify Compound Summary Report   MassLynx 4.1 SCN810

Dataset: Untitled

Last Altered: Tuesday, March 17, 2026 14:35:47 China Standard Time
Printed: At Tuesday, March 17, 2026 14:36:05 China Standard Time
By INFORMATICS\user (user)

Method: C:\MSDATA\gongxun.PRO\MethDB\sclareol.mdb 17 Mar 2026 14:26:43
Calibration: 17 Mar 2026 14:35:47

Compound name: sclareol
Coefficient of Determination: R^2 = 0.999433
Calibration curve: 0.301106 * x
Response type: External Std, Area
Curve type: Linear, Origin: Force, Weighting: 1/x, Axis trans: None

	#	Name	Type	Std. Conc	RT	Area	IS Area	Response	Primar...	ng/ml	%Dev
1	1	std5	Standard	1200.000	1.97	367.827		367.827	bb	1221.6	1.8
2	2	std6	Standard	2000.000	1.97	606.638		606.638	bb	2014.7	0.7
3	3	std7	Standard	4000.000	1.97	1193.501		1193.501	bb	3963.7	-0.9
4	4	DSL-gan3	Analyte		2.03	989.086		989.086	bb	3284.8	
5	5	DSL-xian3	Analyte		1.94	1191.956		1191.956	bb	3958.6	

Quantify Calibration Report   MassLynx 4.1 SCN810

Dataset: Untitled

Last Altered: Tuesday, March 17, 2026 14:35:47 China Standard Time
Printed: At Tuesday, March 17, 2026 14:36:05 China Standard Time
By INFORMATICS\user (user)

Method: C:\MSDATA\gongxun.PRO\MethDB\sclareol.mdb 17 Mar 2026 14:26:43
Calibration: 17 Mar 2026 14:35:47

Compound name: sclareol
Coefficient of Determination: R^2 = 0.999433
Calibration curve: 0.301106 * x
Response type: External Std, Area
Curve type: Linear, Origin: Force, Weighting: 1/x, Axis trans: None

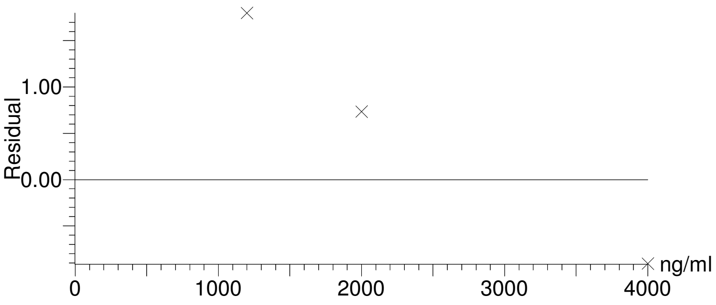

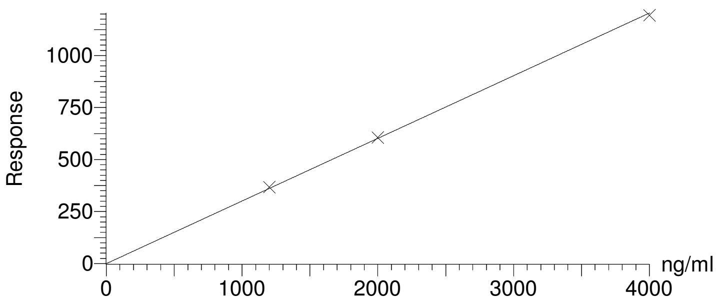

Quantify Compound Report MassLynx 4.1 SCN810

Dataset: Untitled

Last Altered: Tuesday, March 17, 2026 14:35:47 China Standard Time
Printed: At Tuesday, March 17, 2026 14:36:05 China Standard Time
By INFORMATICS\user (user)

Method: C:\MSDATA\gongxun.PRO\MethDB\sclareol.mdb 17 Mar 2026 14:26:43
Calibration: 17 Mar 2026 14:35:47

Sample Name: std5

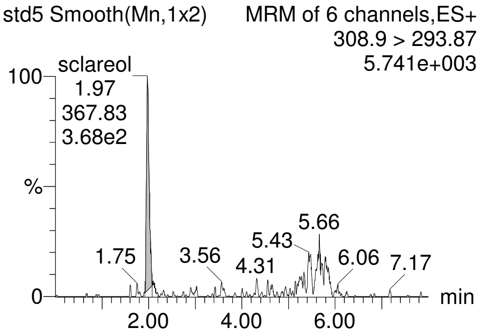

Sample Name: std6

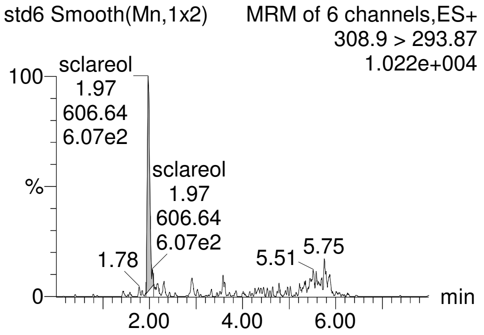

Sample Name: std7

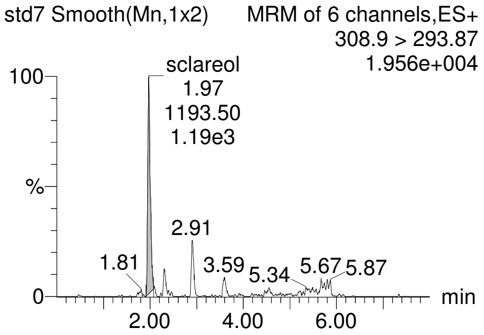

Sample Name: DSL-gan3

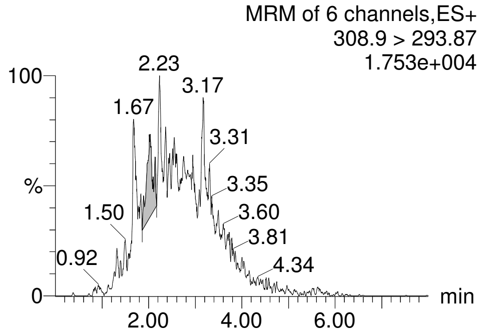

Sample Name: DSL-xian3

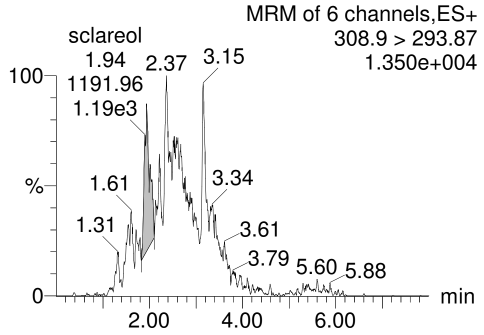

	#	Name	Type	Std. Conc	RT	Area	IS Area	Response	Primar...	ng/ml	%Dev
1	1	std5	Standard	1200.000	1.97	367.827		367.827	bb	1221.6	1.8
2	2	std6	Standard	2000.000	1.97	606.638		606.638	bb	2014.7	0.7
3	3	std7	Standard	4000.000	1.97	1193.501		1193.501	bb	3963.7	-0.9
4	4	DSL-gan3	Analyte		2.03	989.086		989.086	bb	3284.8	
5	5	DSL-xian3	Analyte		1.94	1191.956		1191.956	bb	3958.6	
